# Interactive Effects of Warming and Competition Do Not Limit the Adaptive Plastic Response to Drought in Populations of a Mediterranean Plant

**DOI:** 10.1111/gcb.70363

**Published:** 2025-07-25

**Authors:** Marina Ramos‐Muñoz, Mario Blanco‐Sánchez, Beatriz Pías, José Alberto Ramírez‐Valiente, Raquel Benavides, Adrián Escudero, Silvia Matesanz

**Affiliations:** ^1^ Área de Biodiversidad y Conservación Universidad Rey Juan Carlos Móstoles Spain; ^2^ Instituto de Investigación en Cambio Global (IICG‐URJC) Universidad Rey Juan Carlos Móstoles Spain; ^3^ Department of Terrestrial Ecology Netherlands Institute of Ecology (NIOO‐KNAW) Wageningen the Netherlands; ^4^ Departamento de Biodiversidad, Ecología y Evolución Universidad Complutense de Madrid Madrid Spain; ^5^ ICIFOR‐CN INIA CSIC Madrid Spain; ^6^ Department of Natural Systems and Resources ETSI Montes, Forestal y del Medio Natural Universidad Politécnica de Madrid Madrid Spain; ^7^ Centro Para la Conservación de la Biodiversidad y el Desarrollo Sostenible Universidad Politécnica de Madrid Madrid Spain

**Keywords:** antagonism, drought, interacting stressors, intraspecific competition, multivariate environments, phenotypic plasticity, synergy, warming

## Abstract

Water scarcity is the main selective pressure determining the performance of Mediterranean plant populations, with climate change predicted to increase the intensity and duration of droughts. However, drought rarely acts in isolation. Climate change also involves substantial warming in this region and may disrupt natural processes, including biotic interactions. Phenotypic plasticity allows plants to cope with rapid and multifaceted environmental changes. Although our knowledge of plastic responses to drought in Mediterranean plants has increased in recent years, how co‐occurring simultaneous stressors interact to produce additive, synergistic, or antagonistic effects that enhance or constrain adaptive plastic responses to drought is still unknown. Using a factorial experimental approach based on a multivariate common garden, we assessed whether adaptive phenotypic plasticity to drought and population differentiation in traits related to drought response were affected by the occurrence of other key simultaneous stressors, warming and intraspecific competition, in a Mediterranean gypsum endemic shrub. In response to drought, plants expressed adaptive plastic responses associated with a mixed resource‐use strategy, combining conservative (sclerophyllous leaves with higher water use efficiency) and acquisitive (advanced phenology) phenotypic responses. Although the response to drought was modified by synergistic and antagonistic interactions with warming and competition, these interactions did not change the direction or reduce the extent of adaptive plasticity to drought. This suggests that plastic responses to drought may also provide benefits against warming and competition. Finally, we detected significant population differentiation in all functional traits, but phenotypic differences in reproductive biomass were significantly reduced under combined drought and warming. Our results emphasize the robustness of adaptive plasticity to drought under complex stress scenarios and underscore the importance of realistic, multifactorial experimental approaches to predict whether adaptive responses of plant populations will remain effective in a climate change context and influence their future ecological and evolutionary dynamics.

## Graphical abstract

Plants can modify their phenotype (i.e., the characteristics of an individual) in response to environmental changes, a phenomenon termed phenotypic plasticity. Adaptive plasticity may maintain or increase individual fitness, being crucial to cope with complex environmental changes. Although drought is the main selective pressure for Mediterranean plants, other simultaneous stressors may limit adaptive plastic responses, compromising the persistence of plant populations. Our results showed that adaptive plasticity to drought is not constrained by warming or intraspecific competition in a Mediterranean semiarid shrub, highlighting the role of phenotypic plasticity to cope with climate change.

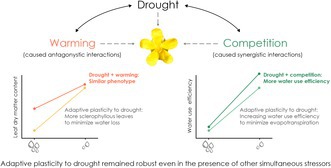

## 1 Introduction

In Mediterranean ecosystems, water availability is both limited and highly variable, and consequently drought is a major selective pressure for plant populations (Lionello 2012). Drought events have become more intense and frequent over recent decades and are projected to further intensify due to climate change, highlighting the urgent need to understand plant responses to complex and realistic environments (Giorgi and Lionello 2008; Viceto et al. 2019). However, drought seldom acts in isolation. Recent climatic projections for the Mediterranean region also forecast general warming, with substantial spatiotemporal variation in temperature patterns (Giorgi and Lionello 2008; IPCC 2023). Consequently, parts of the Mediterranean will likely experience extreme temperature events such as heatwaves and substantial reductions in precipitation simultaneously (Giorgi and Lionello 2008; Zittis et al. 2022). Climate change drivers may also disrupt natural processes, including biotic interactions such as inter‐ and intraspecific competition, herbivory, and pathogen dynamics. Biotic interactions may directly affect plant fitness but also influence plant responses to drought (Gallego‐Tévar et al. 2024; Lorts and Lasky 2020). Therefore, predicting how Mediterranean plant populations will respond to climate change and their long‐term survival requires understanding plant responses to drought within multivariate environmental contexts.

Plant populations can respond to such new environments through a complex combination of migration and in situ evolutionary processes (i.e., adaptive evolution and phenotypic plasticity; Franks et al. 2014; Jump and Peñuelas 2005). In situ responses may be particularly relevant for species with limited seed dispersal and fragmented distributions. Within these processes, phenotypic plasticity can provide rapid, adaptive phenotypic variation to cope with novel environmental conditions (Blanco‐Sánchez et al. 2023; Fox et al. 2019). Phenotypic plasticity, the ability of a genotype to change the phenotype in different environments, is a key mechanism to cope with heterogeneous and changing environmental conditions (Fox et al. 2019; Pigliucci 2001; Valladares et al. 2007). Previous studies have shown the ability of Mediterranean plants to respond to drought through plastic responses that often lead to stress‐tolerant phenotypes with sclerophyllous leaves, high water use efficiency, and low photosynthetic and growth rates (Gimeno et al. 2009; Ramírez‐Valiente et al. 2022; Solé‐Medina et al. 2022). However, recent studies have also shown that drought can trigger plastic responses associated with both acquisitive and conservative resource‐use strategies simultaneously (Berger and Ludwig 2014; Blanco‐Sánchez et al. 2023, 2024; Ramos‐Muñoz, Castellanos, et al. 2024). Yet, it remains unclear whether these drought‐induced responses remain unaffected when plants face additional stressors that may amplify or constrain plasticity, and whether such plastic responses still align with adaptive plasticity in more complex, multivariate environments. For instance, warming often induces resource‐use acquisitive phenotypes associated with higher growth rates (Llorens et al. 2004; Yang et al. 2020) and may promote an increase in transpiration rates as a mechanism to cool down leaf temperature (López et al. 2022). These responses to increasing temperatures may conflict with the documented stress‐tolerant responses to drought. Consequently, combined stressors may amplify, mask, or constrain adaptive plastic responses to drought, reshaping both the extent and nature of adaptive strategies under multivariate environments.

**FIGURE 1:**
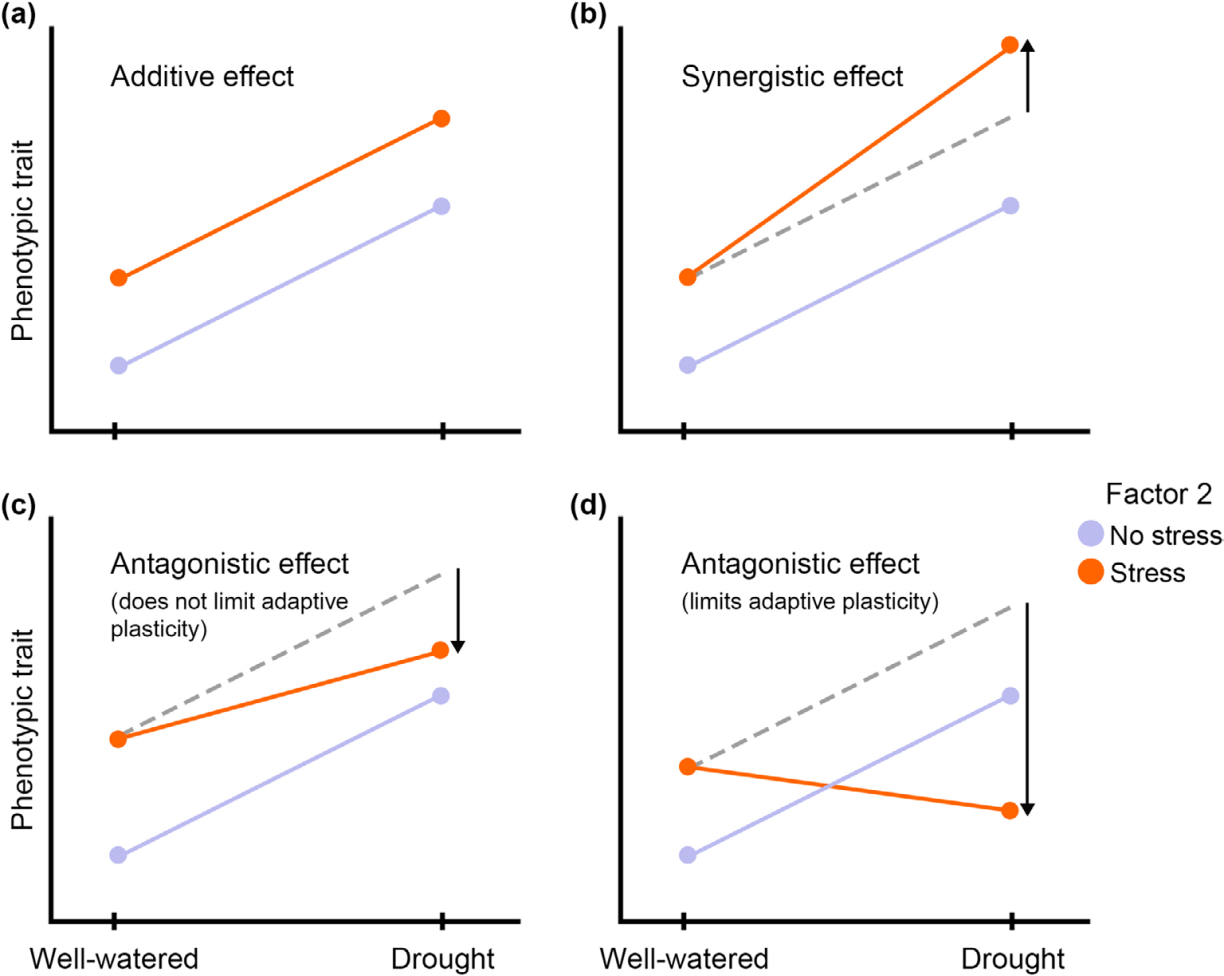
Different scenarios illustrating how adaptive plastic responses to drought (environmental factor Water Availability with two levels in the *x*‐axis) might be modified by the presence of a concurrent environmental factor (Factor 2), i.e., environment‐by‐environment interaction (*E* × *E*). Norms of reaction in lilac represent adaptive responses to water availability for a given functional trait, while potential responses to water availability under the simultaneous presence of a second stressor are shown in orange. To facilitate interpretation, grey dashed lines in panels (b–d) represent theoretical additive effects (no *E* × *E*), and both adaptive responses across conditions and adaptive phenotypic values within conditions remain constant (in this example, higher phenotypic values are always adaptive to cope with a given stressor). (a) Additive effects: both factors have significant main effects but no *E* × *E*. In this case, the magnitude of the adaptive response to drought is not modified by the presence of another stressor. (b) Synergistic interaction between environmental factors. The magnitude of the adaptive response to drought is increased by the presence of a second stressor. (c) Antagonistic interaction between factors that does not limit adaptive responses to drought. The magnitude of the response is lower compared to an additive response, but the phenotype expressed under the combination of stressors is similar to the phenotype expressed under drought alone. (d) Antagonistic interaction between factors that limits adaptive responses to drought. The presence of the second stressor triggers a response in the opposite direction of adaptive responses, and the resulting phenotype under more stressful conditions is less adaptive.

Simultaneous stressors can trigger complex and unpredictable responses with additive, synergistic, or antagonistic effects on plant phenotypes (Côté et al. 2016; Zandalinas and Mittler 2022). Additive effects occur when there is no interaction between stressors, and the total effect is equal to their sum (Figure 1a). Synergistic interactions happen when the combined effect of stressors is greater than the sum of individual effects. While synergistic effects on performance traits typically intensify stress impacts (e.g., excessively reducing individual fitness), they can also amplify the plastic response of other functional traits (Figure 1b). In contrast, antagonistic interactions occur when the combined effect is smaller than the sum of individual effects (Figure 1c,d). In some cases, these interactions can even reverse the response to a particular stressor, ultimately changing the magnitude of adaptive plasticity if the plastic response to drought is advantageous under combined stressors (Figure 1d). For instance, a well‐documented adaptive response to drought is reducing specific leaf area (SLA) to minimize water loss (Ramírez‐Valiente et al. 2010), but competition often triggers higher SLA (Lorts and Lasky 2020), which may compromise the adaptive response to drought. This example illustrates the need to determine if the presence of other stressors modifies or constrains drought‐induced plastic responses in a way that maintains or limits their adaptive value.

Although assessing how interactions between stressors shape plastic responses is key in a climate change context, most studies focus on responses to a particular stressor or the additive effects of combined stressors (Rillig et al. 2019; Speißer et al. 2022), often neglecting fully factorial experiments in which the nature of interactions can be assessed. Furthermore, when interactions are in fact evaluated, the focus is mainly on the effects on performance traits (Homet et al. 2023; Hudak and Dybdahl 2023; Zandalinas and Mittler 2022). However, whether adaptive plasticity can be maintained without being compromised by antagonistic interactions with other stressors remains largely unexplored (Homet et al. 2023; Hudak and Dybdahl 2023; Lorts and Lasky 2020), which has led to an incomplete understanding of how phenotypic plasticity operates under multivariate, realistic conditions.

While many studies addressing interactions among factors have focused on species‐level effects, community dynamics, or even ecosystem functions, very few have assessed how multiple stressors impact population‐level plasticity patterns and population differentiation (Côté et al. 2016; Ravaglioli et al. 2017; Speißer et al. 2022; Zandalinas and Mittler 2022). Yet, population differentiation is often modified by environmental conditions, which may lead to different plastic responses to combined stressors. The co‐occurrence of multiple stressors may alter population plasticity patterns differently, potentially altering genetically based population differentiation under highly stressful conditions (Matesanz and Ramírez‐Valiente 2019). Populations with evolutionary histories of exposure to multiple stressors may be better equipped to cope with these conditions and consequently have higher chances of long‐term persistence. Evaluating how multivariate environmental conditions shape both adaptive plastic responses to drought and population differentiation is therefore key to understanding the role of plasticity and to predicting the persistence of plant populations in a changing climate.

The goal of our study was to evaluate how both climate‐driven and biotic stressors, namely, warming and intraspecific competition, shape adaptive plasticity to drought, and whether interactions among stressors differentially affect populations of a Mediterranean shrub. We selected *Helianthemum squamatum* due to its distribution across semiarid Mediterranean ecosystems, where warming and competitive interactions, which vary significantly within and among populations, may intensify the negative effects of the increasing drought. Using a factorial, multivariate common garden, we tested the effects of warming and intraspecific competition on plastic responses to drought and population‐level variation under drought conditions. We measured a wide set of ecophysiological and reproductive fitness traits in individuals from six populations covering the entire environmental range of this species. We hypothesize that warming and competition will interact to produce conflicting responses to drought. While drought is expected to favor resource‐conservative phenotypes, warming and competition, in contrast, may trigger more resource‐acquisitive strategies (Lorts and Lasky 2020; Prieto et al. 2023). These contrasting effects may not only constrain adaptive plasticity to drought in multivariate environments but also alter what constitutes adaptive responses in these new environments. Finally, since highly stressful conditions often lead to phenotypic convergence across populations (Matesanz and Ramírez‐Valiente 2019), we predict that multiple stressors will reduce population differentiation, but populations from harsher sites will show higher performance under such stressful conditions, reflecting stronger historical selection.

## 2 Methods

### 2.1 Study Species and Seed Collection From Natural Populations

*Helianthemum squamatum* (L.) Dum. Cours. (Cistaceae) is a small shrub (10–40 cm) mainly distributed in the gypsum habitats of the eastern half of the Iberian Peninsula (with one locality in northern Algeria; Figure S1; López González 1993; Mota et al. 2011). Gypsum habitats are characterized by a discontinuous spatial distribution, with gypsum outcrops often immersed in other substrates, forming an island‐like configuration (see Figure S1). These habitats support rich plant communities with a high number of endemic species restricted to gypsum soils (i.e., gypsophiles; Escudero et al. 2015; Meyer 1986; Mota et al. 2003) which include *H. squamatum*. The species blooms from May to August, corresponding with the Mediterranean late spring and summer drought (Aragón et al. 2008; López González 1993). Plants produce small, yellow hermaphrodite flowers in racemose inflorescences (Aragón et al. 2007). This species is a non‐clonal, perennial shrub with a shallow‐rooting system, with most roots concentrated in the top 25 cm of soil (Aragón et al. 2009; De la Puente et al. 2024; Guerrero‐Campo et al. 2006). *Helianthemum squamatum* is diploid, mainly outcrosser with partial self‐compatibility and limited seed dispersal (Escudero et al. 1999; Matesanz et al. 2018). In natural conditions, plants of *H. squamatum* often form highly dense patches of conspecific individuals, which is favored by its limited dispersal and the presence of a mucilaginous seed coat that promotes seed adhesion to the soil (Escudero et al. 2005; Quintana‐Ascencio et al. 2009).

*Helianthemum squamatum* is a fitting model to test the effect of combined stressors on adaptive plastic responses to drought and population differentiation for several reasons: (i) It is one of the most representative perennial shrubs and constitutes a diagnostic species for the Iberian gypsum habitats (Aragón and Escudero 2008). Gypsum habitats are established in semiarid conditions, where the effects of climate change are expected to be more intense (Zittis et al. 2022). (ii) Although it flowers during summer, indicating its ability to reproduce under harsh environmental conditions, the predicted increase in aridity can compromise its current reproductive phenology (Blanco‐Sánchez et al. 2022). (iii) Across its natural distribution, there is ample spatiotemporal variation in water availability, temperature, and population density, both among populations (> 4°C and ≈2 fold variation among populations in temperature and precipitation, respectively) as well as across years in the same location (up to 2.5°C interannual temperature variation over a 35‐year period, data extracted from CHELSA time‐series (Karger et al. 2017) for several *H. squamatum* populations). Similarly, the density of conspecific individuals in populations of central Spain significantly varies between 1 and ≈44 individuals/m^2^ (personal data); and (iv) previous research on *H. squamatum* has shown significant declines in reproductive fitness under dry and hot conditions and lower survival in highly dense patches of the species, which suggests that drought, warming, and intraspecific competition are key selection pressures for this species (Escudero et al. 2005; García‐Cervigón et al. 2021; León‐Sánchez et al. 2016). Furthermore, because semiarid Mediterranean areas have been cataloged as a hotspot for climate change (IPCC 2023), the results from this study will provide relevant insights into how Mediterranean plant populations can respond to new multivariate environmental challenges. This combination of regional climate vulnerability and strong edaphic specialization makes gypsum endemics ideal models to investigate the limits and potential of plant responses to climate change.

In mid‐July 2020, we sampled six populations of *H. squamatum* across the Iberian Peninsula, covering the geographical and climatic range of the species (Figure S1). Sampled populations were distributed along a gradient of annual precipitation and mean temperature (291–461.75 mm, 12.4°C–16.5°C, respectively; data extracted from WorldClim bioclimatic variables (Fick and Hijmans 2017) using a 2 km buffer around each population; Table S1). Within each population, we collected mature fruits from 20 different plants, ensuring a minimum distance of 2 m between sampled individuals to avoid closely related plants. Fruits of each individual plant were collected separately in paper bags, and their seeds were later cleaned in the laboratory.

### 2.2 Common Garden Conditions and Experimental Treatments

In early August 2020, we combined seeds collected from each of the 20 maternal plants in equal proportions to create a seed pool for each population. The seeds of *H. squamatum* have physical dormancy, so we mechanically scarified them with 500 grit sandpaper to enhance germination (Pérez‐García et al. 1995). Scarified seeds were sown in 0.5 L pots with gypsum soil extracted from a nearby gypsum quarry (Yesos Ibéricos‐Algiss S.A., Valdemoro, Madrid, Spain). All pots were maintained well‐watered in the greenhouse of the CULTIVE laboratory at URJC (Móstoles, Madrid, Spain) for ~2 months. To ensure that the used gypsum soil did not contain a seed bank of *H. squamatum*, we set up control pots filled with the same gypsum soil but without sowing any seeds. These pots were maintained under the same conditions and no germination of *H. squamatum* was observed. In early October 2020, pots were randomly thinned to one experimental individual plant, which was transplanted into 6 L plastic pots (Alpifer, Valencia, Spain) filled with the same substrate. Pots were transferred to the outdoor cultivation facilities and maintained under uniform, common well‐watered conditions until the second growth season (spring of 2022), when experimental treatments were applied. This ensured that phenotypic measurements could be taken on adult, reproductive individuals and minimized those maternal effects expressed earlier in the life cycle. The climatic conditions of the outdoor cultivation facilities fall within the range experienced by individuals in natural conditions in typical Mediterranean gypsum habitats (mean annual precipitation and temperature of 395 mm and 14.47°C, respectively; data from WorldClim bioclimatic layers), conferring more realism to our experimental setting.

In early March 2022, individuals were assigned to experimental treatments resulting from the factorial combination of two abiotic factors, each with two levels, representing a non‐stressful and stressful environment: Water availability (Well‐watered/Drought) and Temperature (Warming/No Warming) and one biotic factor, Intraspecific competition (No‐competitor/Competitor). This created a total of eight multifactorial environments. This experimental design mimics an ecologically meaningful stress gradient that recreates natural conditions, with plants growing under higher water availability, without warming and competition (i.e., the least stressful treatment) to plants subjected to drought, warming conditions, and grown in the presence of a conspecific individual (i.e., combination of three stressors as the most stressful treatment; see Figure S2). Importantly, these experimental factors may vary concomitantly in natural conditions (e.g., drought and warming often co‐occur in Mediterranean ecosystems during late spring and summer; (Vogel et al. 2021)). However, water and temperature‐related stress can also occur independently. In the Mediterranean region, warming events, such as heatwaves, can arise independently of drought conditions, particularly during spring. Previous studies indicate that early heatwaves often occur in April or May, even when adequate precipitation is still present (IPCC 2023). Similarly, the number of conspecifics in natural patches of *Helianthemum squamatum* might be lower in years with harsh climatic conditions, but can also vary independently over time due to natural population dynamics (Aragón et al. 2009; Caballero et al. 2005). Therefore, our orthogonal design allows us to test the effects of variations in abiotic and biotic environmental factors in the response to drought but also their effects in isolation.

**FIGURE 2:**
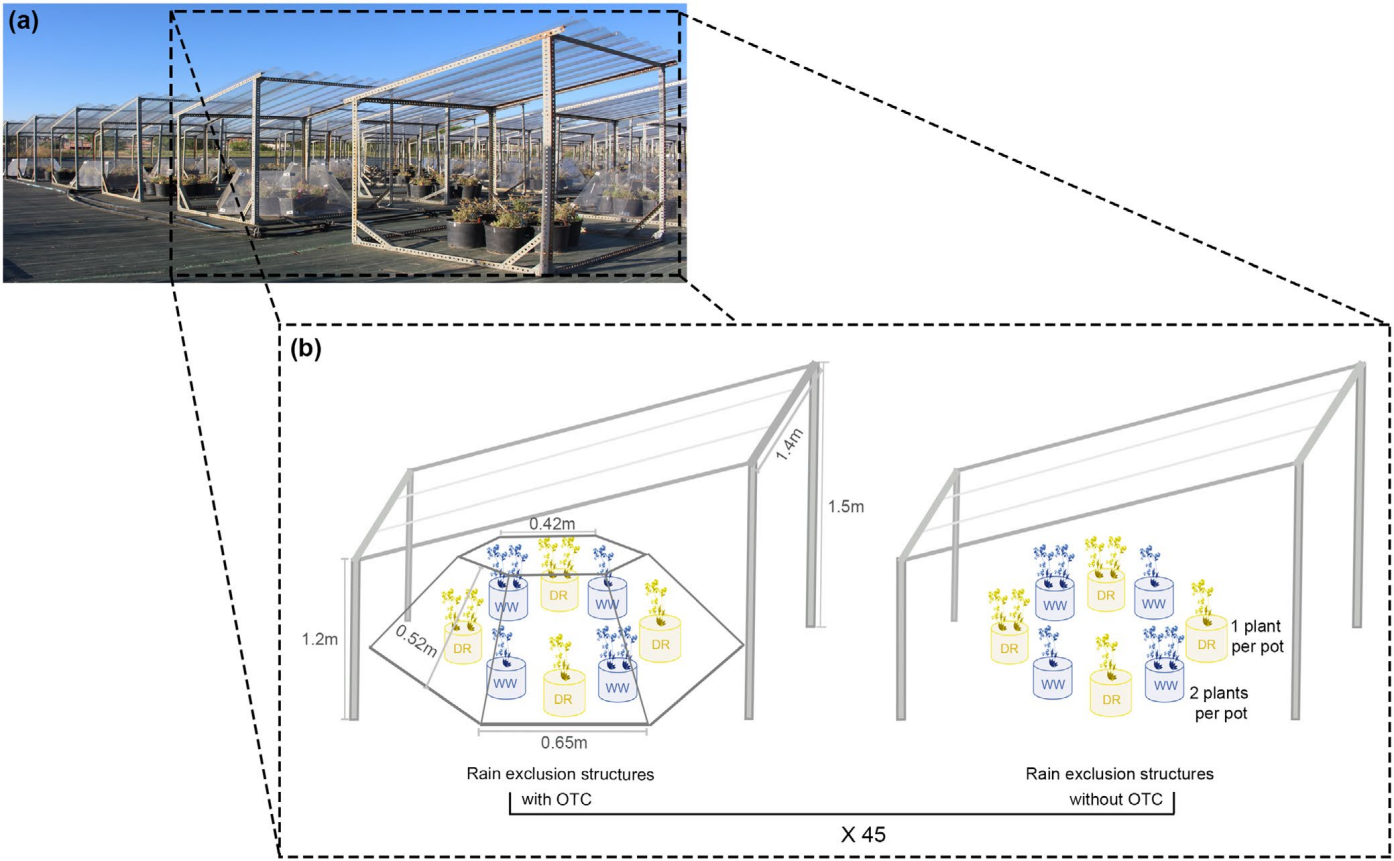
Experimental design of the common garden with multivariate environments in the CULTIVE facilities (URJC, Madrid, Spain). (a) Overview of the common garden experiment using a factorial design of three environmental factors (Water availability, Temperature, and Intraspecific Competition) each with two levels (Well‐watered vs. Drought; No warming vs. Warming; No‐competitor vs. Competitor). The experimental layout consisted of a uniform grid with 90 paired rain‐exclusion structures that removed all‐natural precipitation. The black‐dashed box highlights a representative pair of rain exclusion structures represented in panel (b). (b) Each pair contained one structure with an open‐top chamber (OTC, left part of the diagram) that raised air temperature ~2.4°C and one without, and all eight experimental treatments were represented within each pair. Each structure held eight experimental pots, and half of the experimental individuals were grown alongside a conspecific competitor. Pots in blue and yellow represent the Well‐watered and Drought levels, respectively. The density of conspecific individuals was modified on a pot‐level basis, with one or two plants per pot.

In each treatment, 15 experimental individuals per population were grown, for a total of 720 plants (6 populations × 2 Water availability levels × 2 Temperature levels × 2 Intraspecific competition levels × 15 plants/population/treatment). The experimental layout consisted of a uniform grid with paired rain‐exclusion structures, each pair containing one structure with an open‐top chamber (OTC, see below) and one without, and all eight experimental treatments represented within each pair. A total of 45 pairs of structures were used, providing spatial replication across the entire experimental setup (Figure 2).

*Water availability*. We implemented two levels of Water availability: Well‐watered and Drought. Plants in the well‐watered level were kept at field capacity for our gypsum substrate (~24%–26% soil water content, SWC hereafter; Figure S3), simulating water availability during periods with higher soil moisture (e.g., in early spring) and/or populations with higher precipitation. In contrast, under drought conditions, SWC was gradually reduced and then maintained at ~50% of field capacity (~12%–14% for our gypsum soil; Figure S3). Drought conditions simulated water availability currently occurring in dry populations and/or periods such as late spring and summer (based on point‐in‐time measurements under field conditions; data not shown), while also mimicking conditions predicted under climate change scenarios for the Mediterranean region, where drought will be more frequent and intense (IPCC 2023).

To eliminate natural precipitation and ensure robust control over water availability, pots were moved below rain exclusion structures (eight pots per structure; Figure 2 and Figure S2). Rain exclusion structures were built with steel frameworks topped with corrugated polycarbonate sheets (Rooflite, Wetherill Park, Australia). The characteristics of these structures (1.5 m of height on the largest side and transparent roof; Figure 2 and Figure S2) ensured minimal impact on temperature (average temperature 19.97°C and 19.12°C below and outside the structures, respectively), relative humidity (52.27% and 52.25%), and photosynthetic active radiation (PAR) (maximum PAR 2136 μmol/m^2^/s and 2108 μmol/m^2^/s) compared to outside conditions. The amount of water supplied in each pot was controlled by using pressure‐compensating emitters (Rain Bird XB05PC; Rain Bird Corporation, CA, USA) and modifying the number and duration of watering events throughout the experiment. The same amount of water was applied to all treatments within the same Water availability level (e.g., all treatments assigned to the Drought level received the same amount of water across all combinations involving Drought).

*Temperature*. We manipulated temperature settings at two different levels: No warming and Warming. Temperature in the warming level was increased using OTCs, which are commonly employed in climate change experiments (e.g., Bokhorst et al. 2012; Henry and Molau 1997; Jerome et al. 2021). The increase in average temperature caused by our experimental OTCs (approximately 2.4°C on average, Figure S4) mimics conditions of populations with higher temperatures in the distribution range of the species and matches the predicted values of the latest climate change models for the end of the century under scenarios of moderate emissions (SSP2‐4.5; IPCC 2023). To better characterize the microclimatic conditions generated by the warming treatment, we also calculated the vapor pressure deficit (VPD; Campbell and Norman 2000) using temperature and relative humidity data recorded by HOBO sensors (see below). Mean VPD was 2.58 ± 2.59 kPa under OTC conditions and 2.02 ± 1.91 kPa outside OTCs (Figure S4). OTCs consisted of a hexagonal chamber built of methacrylate plates (six trapezes of 65 × 42 × 52 cm, long side, short side and height, respectively; Figures 2 and Figure S2). The used methacrylate plates have a high transmittance in the visible spectrum (92%), very low emission of the infrared wavelength (4%) and high energy transmission (85%; data provided by the manufacturer, Cholloplastic, Alicante, Spain). OTCs were raised 5 cm from the soil surface, which allowed adequate airflow and prevented excessive heating (Figure 2 and Figure S2).

*Intraspecific competition*. Under natural conditions, intraspecific competition frequently causes stressful conditions for plants, since competitors often limit the availability of water and soil nutrients (Aschehoug et al. 2016). Previous evidence indicates lower performance of *H. squamatum* in denser patches of conspecifics (Blanco‐Sánchez et al. 2022; Escudero et al. 1999, 2005). Therefore, to simulate contrasting densities of conspecific individuals that *H. squamatum* encounters under natural conditions, we implemented two levels of Intraspecific competition: No competitor and Competitor. Plants assigned to the Competitor level were grown with another conspecific (i.e., two plants per pot, approx. 44 individuals per m^2^; Figure 2 and Figure S2), simulating dense patches in natural conditions. In contrast, experimental individuals assigned to the No competitor level grew individually (Figure 2 and Figure S2). Seeds that gave rise to the intraspecific competitors were collected from a single, common population in the central Iberian Peninsula (Yebra, Guadalajara), ensuring a uniform competition intensity across all experimental individuals by using competitors with similar phenotypes. This population was chosen for its intermediate climate conditions within the range of the experimental populations (Table S1). Competitors were transplanted into the 6 L pots at the same time as the experimental individuals, and all competitors were placed at approximately equal distance to the experimental individuals, which was measured after transplant.

Air temperature and relative humidity were monitored every 10 min across experimental treatments by using 10 automated sensors (HOBO U23 Pro v.2 Temp/RH, Onset Corporation, Bourne, MA, USA; Figure S4) placed randomly throughout the experimental set‐up, in five pairs of rain exclusion structures with and without OTC (Figure 2), while PAR was recorded both below and outside rain exclusion structures with two meteorological stations (HOBO H21‐USB, Onset Corporation, Bourna, MA, USA). To monitor water availability, we measured SWC three times per week in 10–12 pots in each experimental treatment using a HH2 Moisture Meter (Delta‐T Devices, Cambridge, UK; see Figure S3).

### 2.3 Measurements of Functional Traits

We measured leaf morphology, physiology, phenology, and growth traits related to drought response, as well as reproductive fitness in all plants.

At the reproductive peak of *H. squamatum* (end of June 2022), physiological and leaf morphological traits were measured. Specifically, we measured midday photochemical efficiency (*F*_*v*_ */F_m_*) as a proxy of photosynthetic efficiency with a portable pulse‐modulated fluorimeter (Handy PEA+ chlorophyll fluorimeter, Hansatech, UK). These measurements were taken from 13:00 to 16:00 h (UTC + 2, during two consecutive sunny days) in one fully developed and healthy leaf previously adapted to dark with a leaf clip for 30 min, to ensure that all PSII centers were opened (Maxwell and Johnson 2000). Then, we selected five fully developed and healthy leaves per plant, stored them in plastic bags filled with water‐saturated filter paper, and placed them overnight in cool and dark conditions (at 4°C in the fridge) to ensure leaf rehydration. After ~12 h, water‐saturated leaves were weighed using a Mettler Toledo MX5 microbalance (1 μg precision; Mettler Toledo, Columbus, OH, USA), scanned using an Epson Perfection V370 Photo scanner (Seiko Epson Corporation, Tokyo, Japan) and leaf area was assessed using Adobe Photoshop (Adobe Systems Inc., CA, USA). Then, leaves were oven dried for 48 h at 60°C and weighed again using the microbalance to obtain their oven‐dry mass. Specific leaf area (SLA) was calculated as the ratio of the one‐side area of fresh leaves divided by the oven‐dry mass per individual plant, and leaf dry matter content (LDMC) as the dry mass of leaves divided by their water‐saturated mass (Cornelissen et al. 2003).

The presence of at least one inflorescence with open flowers was monitored in all plants four to five times per week throughout the experiment to determine the flowering onset (FO) for each plant. At the end of the reproductive season, in July, we counted the number of viable inflorescences of each plant, and we randomly collected three mature inflorescences per plant. At the lab, we dissected these three inflorescences and counted the number of viable seeds per inflorescence to calculate the mean number of viable seeds per inflorescence. Furthermore, 10 viable seeds per plant were randomly selected and individually weighed using a microbalance to obtain the mean individual seed mass (ISM) of each plant. From these measurements, we determined total seed mass (TSM) as an integrated variable of reproductive fitness, calculated as the product between the number of viable seeds per inflorescence, the number of viable inflorescences, and ISM of each plant.

Finally, we also measured the height, maximum radius, and orthogonal radius to the maximum one in all plants at the beginning and end of the experiment (mid‐March and early July, respectively). With these measurements, we calculated the initial and final volume of each plant as $\frac{2}{3}\;\pi \cdot r_{1}\cdot r_{2}\cdot h$ (*r*_1_ and *r*_2_ are the measured radii and h is the height of the plant) to subsequently estimate the relative growth rate (RGR) as $\frac{\left(\ln\;V_{2}-\ln\;V_{1}\right)}{T_{2-1}}$ where *V*_1_ and *V*_2_ are initial and final volume, respectively, and *T*_2‐1_ is 133 days, the number of days elapsed between the two measurements. At the end of the experiment, reproductive biomass (RB) was collected and weighed in a Kern ABJ 120‐4 M analytical balance (1 mg precision; Kern and Sohn GmbH, Albstadt, Germany). Finally, we determined the leaf carbon (% C) and nitrogen content (% N) and carbon and nitrogen stable isotope ratios (δ^13^C and δ^15^N) as proxies of water use efficiency (WUE) and N assimilation, respectively (Ariz et al. 2015; Robinson et al. 2000), in 12 plants per population and treatment (*N* = 572). These analyses were performed at the Stable Isotopes and Instrumental Analysis Facility in Universidade de Lisboa (Lisbon, Portugal).

### 2.4 Statistical Analyses

To test the effect of Water availability (Well‐watered vs. Drought; Environment, *E*) on phenotypic expression, assess how Temperature (Warming vs. No Warming) and Intraspecific competition (Competitor vs. No Competitor) influenced these responses (*E* × *E*), and whether these effects differed among populations (P, *P* × *E* and *P* × *E* × *E*), we used linear mixed models with restricted maximum likelihood (REML) for each phenotypic trait. Our models included each phenotypic trait as the response variable, with the main effects, two‐way, three‐way, and four‐way interactions of Population, Water availability, Temperature, and Intraspecific competition as explanatory variables. To account for potential spatial autocorrelation of the microenvironmental conditions within our experiment, the location of each rain exclusion structure pair was included as a random factor. Statistical models that included the distance between the focal plant and the competitor as a covariate to account for potential distance‐related effects on the phenotype provided almost identical results (data not shown). Response variables were square‐root or logarithm‐transformed when necessary to meet assumptions of normality and homoscedasticity.

Significance of fixed factors was tested using function Anova (package car; Fox and Weisberg 2011), with type III sum of squares and the Kenward–Roger approximation to calculate the residual degrees of freedom. To avoid type I errors related to multiple testing, significance levels for each term of the model were corrected by false discovery rate (FDR; Benjamini and Hochberg 1995) using the p.adjust function. A significant effect of population (P) indicated genetically based phenotypic differentiation among populations. A significant effect of a particular environmental factor (*E*) reflected a plastic response to Water availability, Temperature, or Intraspecific competition. A significant interaction between Water availability and either Temperature or Intraspecific competition indicated that the plastic response to water availability differed depending on the presence of the other stressors, either as a synergistic or antagonistic interaction. Conversely, lack of *E* × *E* interactions indicated the presence of additive effects on plastic responses across conditions (i.e., the effect of combined, multiple stressors is equal to the effect of their sum). Finally, a significant interaction between Population, Water availability, and either Temperature or Intraspecific Competition (*P* × *E* × *E*) indicated that population‐level plastic responses to drought were differentially affected by the presence of warming or intraspecific competition (non‐parallel norms of reaction among populations), affecting in turn population differentiation within environments.

When a significant *P* × *E* interaction was found, Tukey post hoc tests were used to assess phenotypic differentiation among populations within levels of an environmental factor. Furthermore, when a significant *E* × *E* interaction was found between Water availability and either Temperature or Intraspecific Competition, Tukey post hoc tests were used to assess phenotypic differentiation between levels of the second factor within each level of Water availability. When a significant triple interaction was found (i.e., *P* × *E* × *E*), data were subset by the levels of Temperature and Intraspecific competition, and then Tukey post hoc tests were performed to assess phenotypic differentiation among populations within the levels of Water availability. In all models, marginal and conditional R^2^, the variance explained by only fixed factors and by both fixed and random factors, respectively, were calculated using r.squaredGLMM function (package MuMIn; Barton 2020).

## 3 Results

Nearly all the traits evaluated exhibited significant phenotypic plasticity to Water availability, Temperature and Intraspecific competition (Table S2). The response to Water availability was modified by interactions with Temperature and Intraspecific competition, leading to synergistic and antagonistic effects on several traits. Furthermore, there was significant population differentiation for all functional traits, and population differentiation was reduced under combined drought and warming conditions (Table S2). Neither the triple interaction between environmental factors nor the quadruple interaction between these factors and Population (*W* × *T* × *C* and *P* × *W* × *T* × *C* in Table S2, respectively) were significant for any of the traits studied. Conditional R^2^ was only slightly higher than marginal R^2^ in all the traits evaluated, indicating that the phenotype of experimental individuals was not substantially affected by their position across our common garden setting.

### 3.1 Plastic Responses to Environmental Factors

**FIGURE 3:**
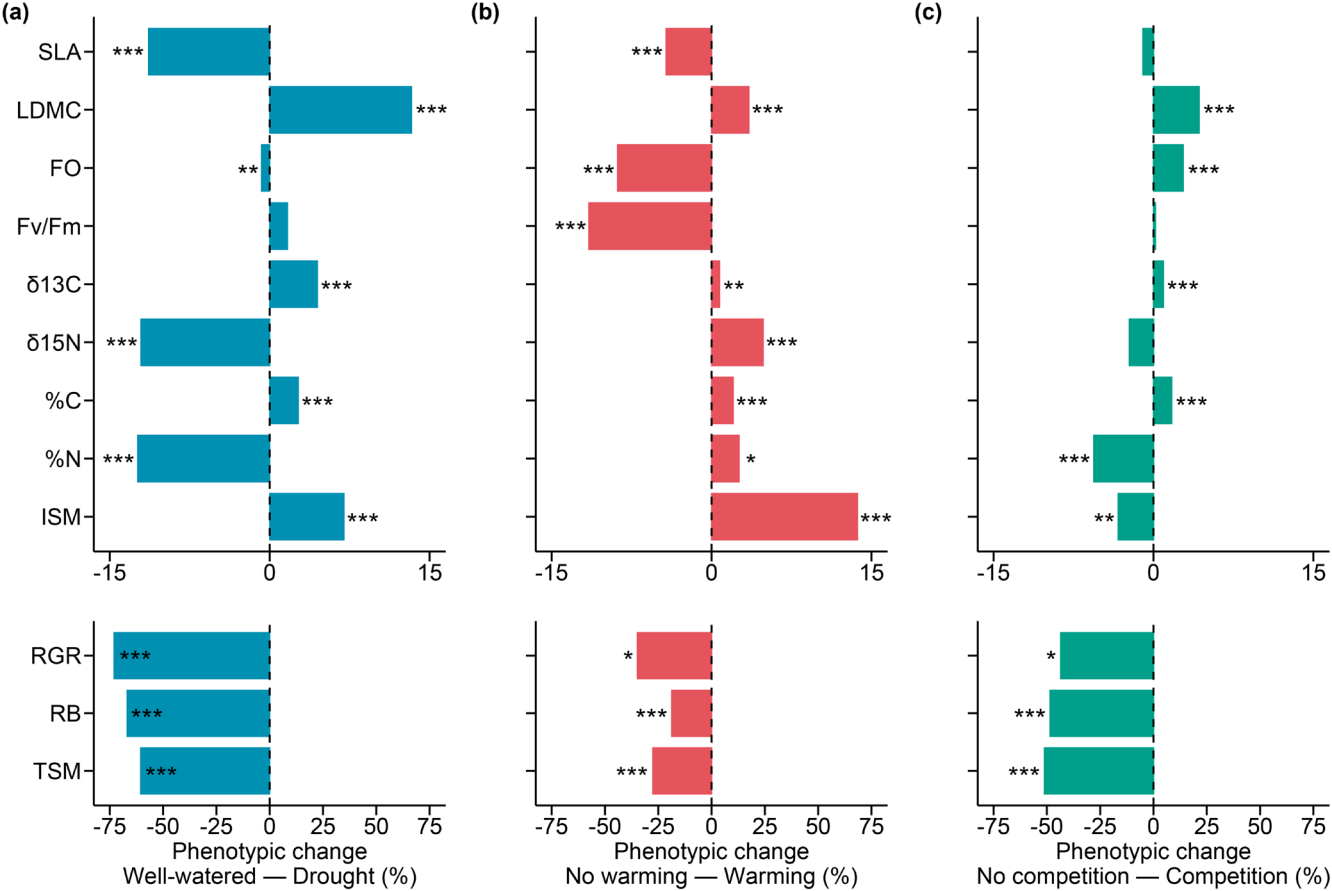
Percentage of phenotypic change between levels of (a) Water availability, (b) Temperature, and (c) Intraspecific competition. Each bar represents the relative change in trait values using the non‐stressful level of each environmental factor (Well‐watered, No warming and No‐competitor) as the reference. For example, a negative value in panel (a) indicates an overall decrease in a trait when comparing plants grown under drought conditions to those grown under well‐watered conditions across all experimental treatments. These results correspond to the main effects of the environmental factors in Table S2. Note that the *x*‐axis scale for RGR, RB, and TSM differs from that of the other traits to facilitate the visualization. Trait abbreviations: SLA, specific leaf area (cm^2^/g); LDMC, leaf dry matter content (mg/g); FO, flowering onset (days); *F*_v_/*F*_m_, midday photochemical efficiency; δ^13^C, leaf carbon isotope ratio (‰); δ^15^N, leaf nitrogen isotope ratio (‰); %C, leaf carbon content; %N, leaf nitrogen content; RGR, relative growth rate (cm^3^/days); RB, reproductive biomass (g); ISM, individual seed mass (mg); TSM, total seed mass (mg). Significance levels are shown: *p < 0.05; **p < 0.01; ***p < 0.001. See Table S2 for results of linear mixed models for each trait.

We found significant phenotypic differences between levels of all the environmental factors in most of the traits (i.e., phenotypic plasticity, significant effect of Water availability, *W*; Temperature, *T*; and Intraspecific competition, *C* in Table S2). Specifically, plants modified leaf morphology in response to water availability, showing on average lower SLA and higher LDMC under drought compared to well‐watered conditions (Figure 3a). Similar plastic responses were observed in plants exposed to warming, which also showed lower SLA and higher LDMC relative to non‐warmed plants (Figure 3b). In contrast, plants grown in competition increased LDMC, with no effect on SLA (Figure 3c). Leaf chemical composition also responded plastically to all environmental factors, with some traits showing consistent responses. Specifically, δ^13^C and leaf C content (%C) increased under drought, warming, and intraspecific competition. However, the environmental factors differed in their effects on δ^15^N and leaf N content (%N): drought decreased both traits, intraspecific competition had no effect on δ^15^N but reduced %N, and warming increased both δ^15^N and %N compared to plants not exposed to these conditions (Figure 3).

FO advanced under both drought and warming conditions, with warming causing a more pronounced change (~8 day‐advance; Figure 3b). In contrast, plants delayed FO in response to intraspecific competition compared to plants growing alone (Figure 3c). In addition, plants reduced RB, RGR, and TSM under drought, warming, and competition compared to well‐watered, non‐warmed, and no‐competition conditions. Finally, while plants growing under drought and warming increased ISM, they produced lighter seeds when growing with a competitor than without competition (Figure 3).

### 3.2 Effects of Warming and Competition on the Plastic Response to Drought

**FIGURE 4:**
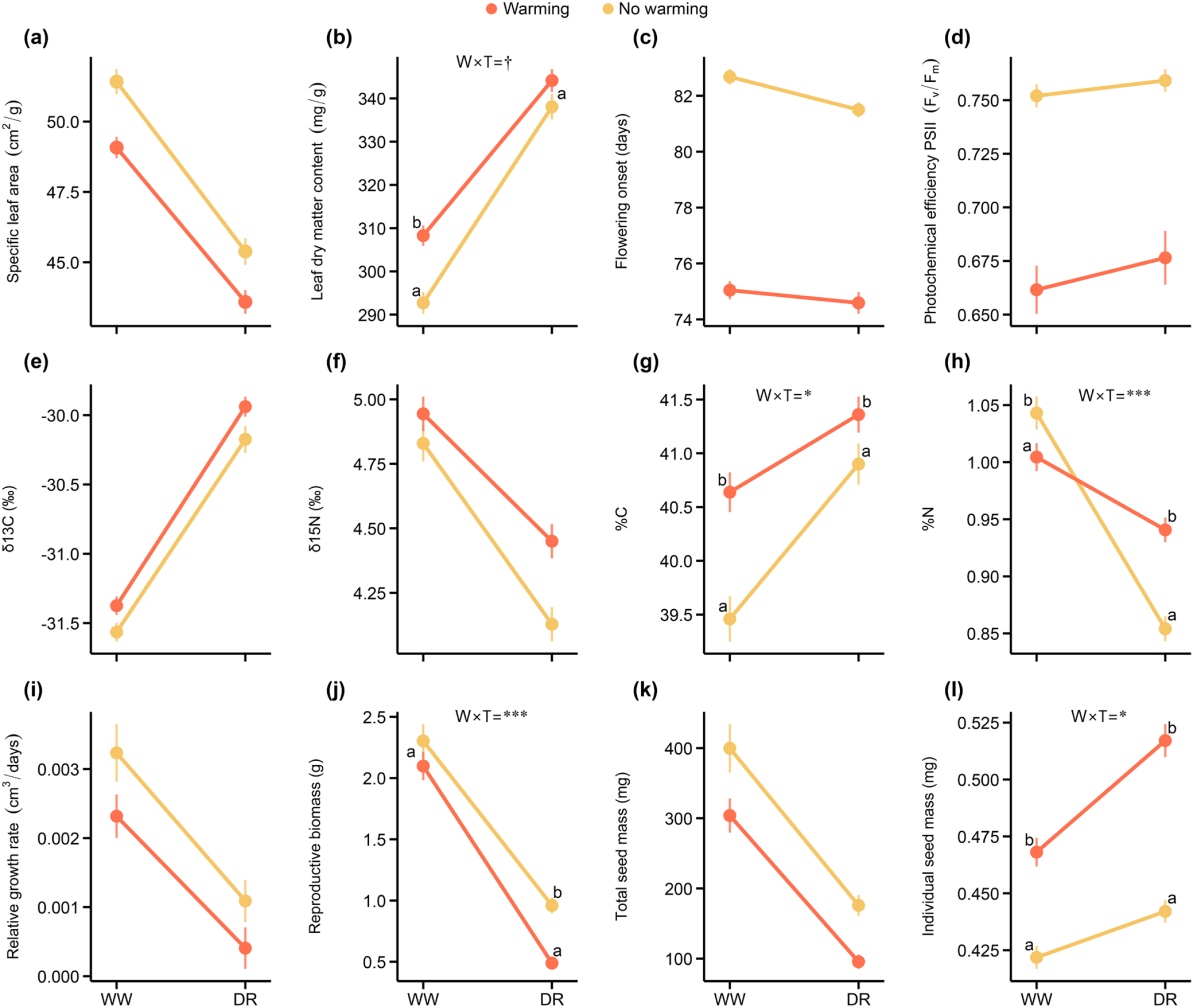
Norms of reaction representing the interaction between Water availability and Temperature (*W* × *T* in Table S2). Phenotypic means (±SE) in each Water availability level (WW: Well‐watered, DR: Drought) and norms of reaction of experimental individuals that experienced warming (in orange) and those that did not (in yellow) are shown. Only significant and marginally significant interactions between Water availability and Temperature are indicated. When a significant or a marginally significant *W* × *T* interaction was found, Tukey's post hoc tests between levels of Temperature factor within each Water availability level were performed. Different letters indicate significant differences between levels of Temperature in mean trait values within each water availability level, according to Tukey's post hoc tests. Significant terms are shown: *p < 0.05; **p < 0.01; ***p < 0.001; while **†** indicates a marginally significant effect (0.05 < *p* < 0.1). See Table S2 for results of linear mixed models for each trait.

Plastic responses to water availability differed depending on the concurrent presence of warming and competition. We detected significant (and marginally significant) double interactions between Water availability and either Temperature or Intraspecific competition for several traits (Table S2), indicating that drought responses were modified by other abiotic and biotic stressors. Specifically, there were significant and marginally significant antagonistic Water availability‐by‐Temperature interactions for LDMC, %C, and %N (Figure 4). Individuals grown under warming conditions had significantly higher LDMC compared to those without warming in well‐watered conditions, but this difference was not significant under drought (Figure 4b). Plants increased %C under warming at both watering levels, but these differences were more pronounced in well‐watered conditions (Figure 4g). Furthermore, under high water availability, individuals exposed to warming had lower %N than those that did not experience warming; whereas under drought, individuals under warming had higher %N than those grown without warming (Figure 4h). In addition, a synergistic interaction resulted in a more pronounced reduction in RB in response to drought under warming compared to no‐warming conditions (Figure 4j). Finally, individuals that experienced warming produced larger seeds (ISM) in both watering levels, but these differences were more pronounced under drought conditions (Figure 4l). In contrast, we did not find a significant Water availability‐by‐Temperature interaction for SLA, FO, *F*_v_/*F*_m_, δ^13^C, δ^15^N, RGR, or TSM, indicating that the effect of Temperature was constant across both levels of Water availability (Table S2 and Figure 4).

**FIGURE 5:**
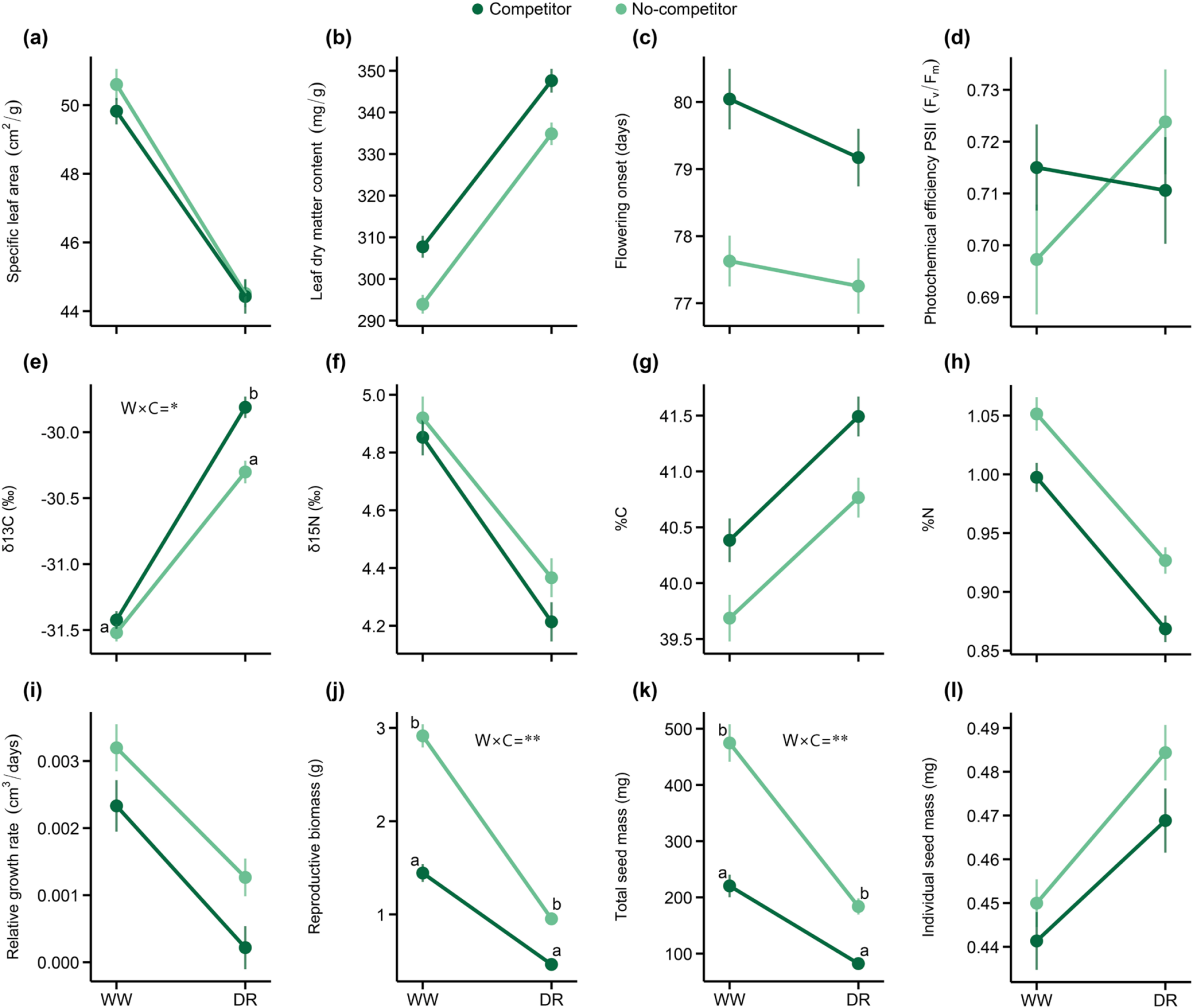
Norms of reaction that represent the interaction between Water availability and Intraspecific competition (*W* × *C* in Table S2). Phenotypic means (±SE) in each Water availability level (WW, well‐watered; DR, drought) and norms of reaction of experimental individuals that experienced intraspecific competition (in dark green) and those without a competitor (in light green) are shown. Only significant and marginally significant interactions between Water availability and Intraspecific competition are indicated. When a significant or a marginally significant *W* × *C* interaction was found, Tukey's post hoc tests between levels of Intraspecific competition factor within each Water availability level were performed. Different letters indicate significant differences between levels of temperature in mean trait values within each water availability level, according to Tukey's post hoc tests. Significant terms are shown: *p < 0.05; **p < 0.01. See Table S2 for the results of linear mixed models for each trait.

We also found both synergistic and antagonistic interactions between Water availability and Intraspecific competition (*W* × *C*). In particular, the interaction between Water availability and Intraspecific competition for δ^13^C was synergistic. Plants increased δ^13^C (i.e., water use efficiency) under drought, with individuals growing with a competitor showing a stronger response than those without it, resulting in significant phenotypic differences only under drought conditions (Figure 5e). In contrast, antagonistic *W* × *C* interactions were observed for reproductive traits. While intraspecific competition reduced RB and TSM across both watering levels, the phenotypic differences between competition levels were smaller under drought conditions, as the fitness reduction in response to drought was higher for individuals grown without a competitor (Figure 5j,k). There were not significant Water availability‐by‐Intraspecific competition interactions for SLA, LDMC, FO, δ^15^N, %C, and ISM, indicating that the effect of Intraspecific competition was constant across both levels of Water availability (Table S2 and Figure 5).

### 3.3 Population Differentiation and Differential Plasticity Across Populations

We detected genetically based phenotypic differences among populations in all traits (significant effect of Population in Table S2). Furthermore, plastic responses were largely similar across populations (not significant *P* × *W*, *P* × *T*, or *P* × *C* interactions for most traits, indicating parallel norms of reaction; Table S2).

**FIGURE 6:**
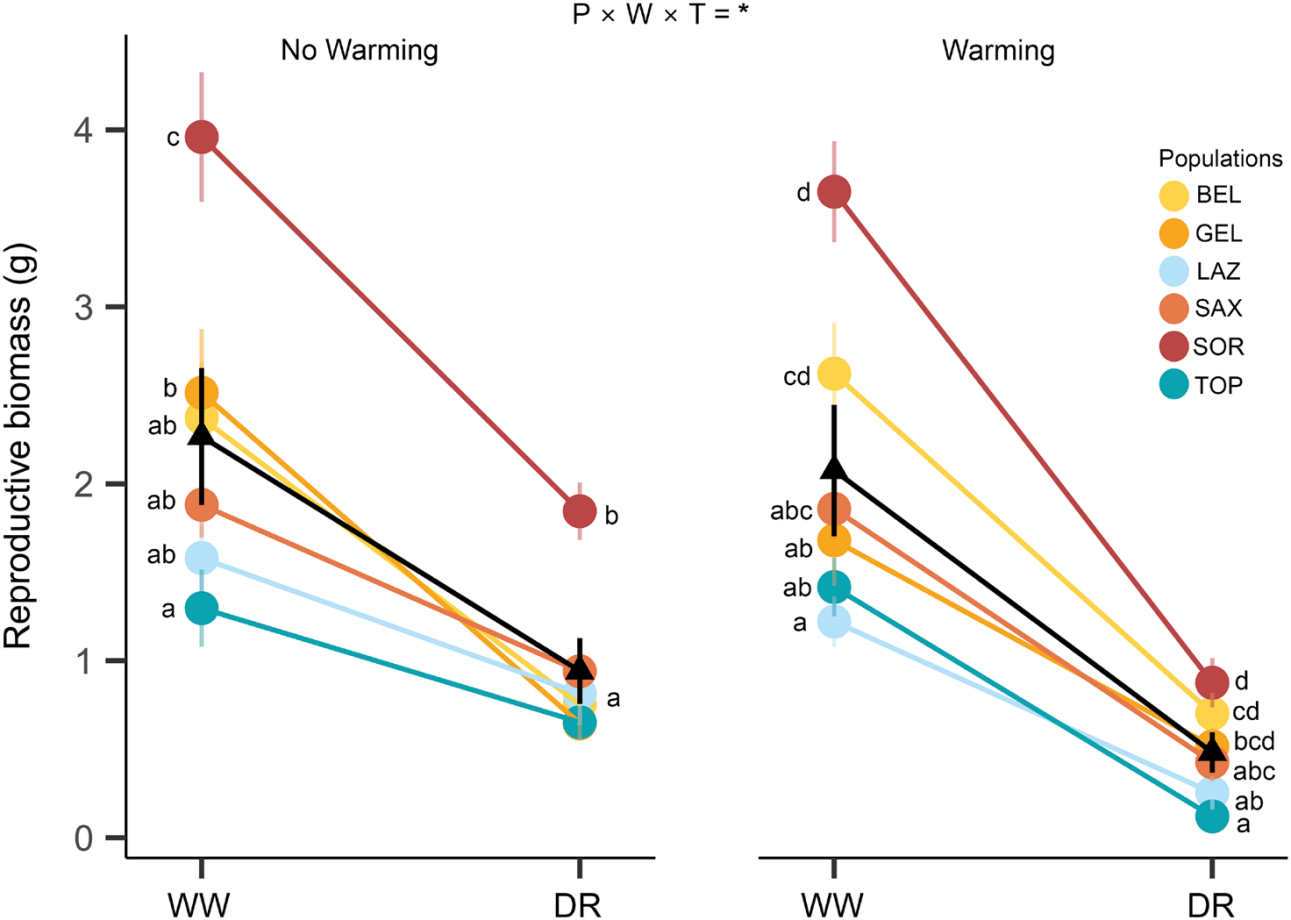
Norms of reaction that represent the significant interaction (*p < 0.05) among Population, Water availability, and Temperature for reproductive biomass (RB). Each colored line shows the norm of reaction of each population and circles show population means (±SE) in each Water availability level (WW, well‐watered; DR, drought). The black line represents the mean norm of reaction of all populations, while black triangles show mean phenotype (±SE) of all populations in each Water availability level. Different letters indicate significant differences among populations in mean trait values within each watering condition, according to Tukey's post hoc tests.

Importantly, we found a significant *P* × *W* × *T* interaction for reproductive fitness (RB) (Table S2 and Figure 6). Population differentiation was reduced when plants experienced both drought and warming compared to drought alone. Specifically, individuals from the SOR population consistently showed higher RB across all environments. However, the differences between SOR and all other populations were smaller under combined drought and warming than under drought alone (Figure 6).

## 4 Discussion

Mediterranean plant populations face multiple stressors, many exacerbated by climate change, with drought as the main selective force (IPCC 2023; Zittis et al. 2022). Future responses will likely involve a complex interplay between migration, adaptive evolution, and phenotypic plasticity. However, limited dispersal and the fast rate of environmental change may make adaptive phenotypic plasticity a particularly key response (Franks et al. 2014; Matesanz et al. 2010; Matesanz and Valladares 2014; Stotz et al. 2021). While phenotypic responses to drought are often explored through univariate experiments, our study emphasizes the importance of multivariate experimental designs to better understand the interactive effects of drought with co‐occurring stressors. Our research on *Helianthemum squamatum* reveals that plastic responses to drought were influenced by warming and intraspecific competition, but none of these interactions constrained adaptive plasticity. Plastic responses remained consistent with adaptive plasticity in the multivariate environment, demonstrating the robustness of the species' response under complex conditions. However, combined stressors reduced population differentiation in reproductive fitness, which may reduce adaptive potential to respond to future environmental changes. The presence of contrasting main effects between experimental factors and the different nature of the interactions found indicated that phenotypic responses to one or a combination of stressors cannot be easily predicted and extrapolated, and should be addressed in realistic multifactorial designs. Importantly, our experimental design allowed us to disentangle the individual and interactive effects of each stressor, providing a clearer understanding of their independent contributions as well as their combined impacts. Our findings provide new insights into the role of adaptive plasticity in coping with the complex challenges imposed by climate change.

### 4.1 Plastic Responses to Drought and Their Adaptive Value

Morphological, phenological, and reproductive traits exhibited plastic responses to water availability, some of which were consistent with adaptive plasticity. Specifically, plants produced more sclerophyllous leaves in response to drought, with lower SLA and higher LDMC and %C. Reductions in SLA and increases in LDMC may be interpreted as adaptive plasticity to drought as they help minimize evapotranspiration (Ramírez‐Valiente et al. 2011; Valladares et al. 2014). Furthermore, leaves with higher %C often show greater structural components such as lignin and hemicellulose, also reducing water loss through a thicker and more resistant cuticle (Niinemets and Tamm 2005). Interestingly, foliar %C values in our study were lower than typically reported for other plant species (see e.g., meta‐analysis by Ma et al. 2018). This may reflect the accumulation of ions in leaves, a known strategy of gypsophiles to tolerate gypsum soil chemistry, which could dilute carbon‐based compounds (Cera et al. 2021; Palacio et al. 2022). Sclerophyllous leaves with higher LDMC and C% are well documented drought adaptations (Capon et al. 2009; Lambrecht et al. 2017), and studies with Mediterranean species, including *H. squamatum*, have also reported plastic changes to a more sclerophyllous syndrome as adaptive responses to drought (Ramírez‐Valiente et al. 2010; Ramos‐Muñoz, Castellanos, et al. 2024).

Experimental plants also exhibited higher WUE (measured by δ^13^C) under drought, indicating a more conservative water‐use strategy. Higher WUE is often associated with sclerophyllous leaves and can enhance survival in water‐limited environments (Blanco‐Sánchez et al. 2023; Solé‐Medina et al. 2022). Plants also advanced their FO under drought conditions (Figure 3), consistent with previous findings in *H. squamatum* and other gypsophiles (Blanco‐Sánchez et al. 2023, 2024; Ramos‐Muñoz, Castellanos, et al. 2024). Early flowering is consistent with a stress‐escape strategy (Volaire 2018) and is adaptive for gypsophiles in natural and experimental conditions, especially when conditions are harsher (Blanco‐Sánchez et al. 2023; Blanco‐Sánchez et al., 2022). While this acquisitive response contrasts with the conservative response found for leaf traits and water use, recent studies show that a mixed strategy with both conservative and acquisitive resource‐use traits might be common in Mediterranean species to cope with high abiotic stress (Blanco‐Sánchez et al. 2024; Kooyers et al. 2015). Our results confirm that exhibiting both acquisitive and conservative trait responses are not mutually exclusive. Interestingly, the combination of relatively short lifespan, high seed production, and specialization to highly stressful soil and climatic conditions in *H. squamatum* suggests that this species may span both stress‐tolerant and ruderal strategies in Grime's CSR framework (Grime 1977; Escudero et al. 2015; Aragón et al. 2009). This mix of traits may underline the coexistence of acquisitive and conservative responses observed under drought.

Finally, plants under drought conditions produced larger seeds (Figure 3), a well‐documented adaptive response that might enhance seedling survival and germination under dry conditions (Blanco‐Sánchez et al. 2024; Germain et al. 2013; Metz et al. 2010). Indeed, both intra‐ and transgenerational adaptive plasticity to drought through increased seed mass has been reported in *H. squamatum* (Ramos‐Muñoz, Blanco‐Sánchez, et al. 2024; Ramos‐Muñoz, Castellanos, et al. 2024).

### 4.2 Interactive Effects of Warming and Competition on Drought Plasticity

Interestingly, some of the adaptive plastic responses to drought were modified by the presence of warming and competition. Warming exerted an antagonistic effect on drought responses, in both LDMC and %C. Individuals exposed to warming had, on average, higher LDMC and %C, but these differences were more pronounced in well‐watered conditions (Figure 4). Warming also increased vapor pressure deficit (VPD, Figure S4), which may have contributed to the observed responses by intensifying atmospheric drought stress. However, these antagonistic interactions did not alter the direction of the response to drought, and the expressed phenotype under both stressors was similar to that under drought alone, suggesting that *H. squamatum* can maintain an adaptive response to drought even under complex environmental conditions.

Similarly, the increase in water use efficiency (WUE) observed in response to drought was enhanced by intraspecific competition through a synergistic interaction (Figure 5). Individuals under drought and competition exhibited higher WUE compared to those exposed to drought alone. Lorts and Lasky (2020) reported higher fitness in plants with higher δ^13^C under both drought and competition, highlighting the adaptive value of increased WUE under more stressful conditions. This aligns with hypotheses predicting that tolerant strategies might be favored over drought escape as stress intensity increases, if conditions become too harsh for rapid resource use (Kooyers et al. 2015; Volaire 2018). Drought also led to a decrease in leaf nitrogen content (N) compared to well‐watered plants (Figure 4). However, individuals exposed to warming had significantly higher leaf N under drought, which represents an antagonistic interaction (Figure 4). In other words, warming buffered the decrease in leaf N produced by drought. While this antagonism might appear to constrain adaptive plasticity, leaf N content reflects multiple ecophysiological processes, which may in turn affect the response of other traits (Anderegg et al. 2018). For instance, higher leaf N often correlates with higher WUE (Bytnerowicz et al. 2023). Therefore, this antagonistic effect might indirectly support adaptive plasticity by enhancing plasticity in other traits critical for drought resistance, such as WUE. Although the underlying mechanisms remain unclear, the higher atmospheric demand (i.e., increased VPD; Figure S4) under warming may have also contributed to changes in nutrient uptake or allocation. These results highlight that coordinated adjustments in the response of several traits may contribute to the maintenance of adaptive plasticity under high stress levels. Finally, plants exposed to combined drought and warming also produced larger seeds than the observed increase in response to drought alone (synergistic interaction; Figure 4), which may be an adaptive response under the predicted drier and warmer conditions in the Mediterranean.

Highly stressful conditions are expected to impose limits on plasticity due to increased costs (Stotz et al. 2021; Valladares et al. 2007; Weinig 2000). Combined stressors may also constrain adaptive plasticity to a particular stress if antagonistic interactions alter the direction or the adaptive value of the plastic response in the multivariate environment (Lorts and Lasky 2020; Valladares et al. 2007). However, in our study, adaptive plasticity to drought remained robust under combined stressors. Warming had antagonistic effects (Figure 4), while competition had synergistic effects (Figure 5e) on trait responses. However, the modified responses remained consistent with adaptive plasticity. Synergistic interactions amplified the plastic response in several traits, whereas antagonistic interactions did not result in phenotypes that differed from that observed under drought conditions alone. This robustness of adaptive plasticity to drought may be explained by two non‐exclusive factors. First, functional responses to a particular stressor may also provide benefits against others (Stotz et al. 2021 and references therein), minimizing the impact of limiting antagonistic interactions. For instance, drought‐induced sclerophyllous leaves with higher WUE aligned with responses to both warming and competition (Figure 3). Second, drought is the primary selective force and the most reliable environmental cue for Mediterranean plant populations, as supported by the highest reduction in fitness caused by drought compared to other stressors (Figure 3). This might reduce the effect of secondary stressors, likely diminishing their ability to override adaptive responses. Our results show that regardless of the type of operating interactions (antagonistic or synergistic) and the nature of the stressors (abiotic vs. biotic), their co‐occurrence did not limit adaptive plasticity to drought in our study species.

### 4.3 Impact of Combined Stressors on Reproductive Fitness and Population Differentiation

Despite the adaptive functional responses, all stressors—especially drought—reduced performance and reproductive fitness (Figure 3). A synergistic interaction was observed between water availability and temperature, while an antagonistic interaction occurred between water availability and intraspecific competition for RB (Figures 4 and 5). Regardless of whether the interaction was synergistic or antagonistic, their ultimate effect was consistent, with a reduction in RB under combined stressors. These findings suggest that multiple stressors impose increased selective pressures that could affect population dynamics in *H. squamatum*.

Interestingly, plasticity patterns were strikingly similar across populations (i.e., no *P* × *E* interaction), and simultaneous stressors did not differentially affect population‐level plastic responses for most traits (i.e., no *P* × *E* × *E* interaction). Previous studies have shown similar plasticity patterns to drought among populations of *H. squamatum* and other dominant Mediterranean gypsophiles (Blanco‐Sánchez et al. 2024; Ramos‐Muñoz, Castellanos, et al. 2024). This suggests that natural selection may have favored convergent plasticity across populations, allowing them to respond similarly to both single and combined stressors (homogenizing selection on plasticity; Pigliucci and Kolodynska 2002). Since environmental heterogeneity has been proposed as a main driver for the evolution of phenotypic plasticity (Kelly 2019; Saltz et al. 2018), these results suggest that similar levels of variation in the studied environmental factors have operated simultaneously in all populations.

Despite the similar plasticity patterns, population differentiation in reproductive biomass (RB) significantly decreased under combined drought and warming (Figure 6 and Table S2). This was mainly due to reduced differences between the driest, warmest population (SOR) and the other populations compared to less restrictive environmental conditions. Under severe stress, we found high phenotypic convergence among populations in reproductive fitness, aligning with previous findings of strong reductions in performance and population differentiation under stress conditions (e.g., Matesanz, Ramos‐Muñoz, Blanco‐Sánchez, et al. 2020, Matesanz, Ramos‐Muñoz, Moncalvillo, et al. 2020; Ramos‐Muñoz, Blanco‐Sánchez, et al. 2024; Ramos‐Muñoz, Castellanos, et al. 2024). Such reductions may be especially pronounced under increasing stress intensity and when multiple, co‐occurring stressors are present (Zandalinas and Mittler 2022), although empirical evidence is still limited, particularly for populations from the most stressful environments within a species' range. Our results highlight the importance of understanding how co‐occurring stressors modify fitness differentiation under complex, realistic environmental conditions (Matesanz and Ramírez‐Valiente 2019), as they provide insights into the future vulnerability of populations. If our experiment had focused solely on water availability, we would have detected a lower fitness decrease in SOR, suggesting higher resilience. Consequently, incorporating other stressors provides a more accurate assessment of population vulnerability, which is especially relevant in the current climate change scenario.

## 5 Conclusions

Our study underscores the importance of common garden experiments that simulate realistic scenarios to better understand how plant populations will respond to climate change. While adaptive plasticity to drought remained robust under multiple stressors, the combined impact of stressors reduced population differentiation and fitness, which could influence population dynamics in the future. These results emphasize the need for more comprehensive experimental approaches to assess plant responses under future climate conditions. Such approaches are essential for developing targeted conservation strategies that address the challenges posed by multiple environmental stressors, ensuring the preservation of biodiversity. Further research considering multiple populations of other Mediterranean species with different ecological and evolutionary backgrounds is needed to assess whether adaptive plasticity to key stressors remains consistently robust under different environmental contexts. Future experiments should also simulate realistic, multivariate conditions to better predict plant persistence under climate change.

## Author Contributions

**Marina Ramos‐Muñoz:** data curation, formal analysis, investigation, methodology, visualization, writing – original draft, writing – review and editing. **Mario Blanco‐Sánchez:** formal analysis, investigation, methodology, visualization, writing – review and editing. **Beatriz Pías:** investigation, methodology, supervision, visualization, writing – review and editing. **José Alberto Ramírez‐Valiente:** investigation, methodology, visualization, writing – review and editing. **Raquel Benavides:** investigation, methodology, visualization, writing – review and editing. **Adrián Escudero:** investigation, methodology, supervision, validation, visualization, writing – review and editing. **Silvia Matesanz:** conceptualization, funding acquisition, investigation, methodology, project administration, supervision, visualization, writing – review and editing.

## Conflicts of Interest

The authors declare no conflicts of interest.

## Supporting information


Data S1.

## Data Availability

The phenotypic data generated during the experiment and the code employed to analyze these data and obtain the findings of this study are openly available in Dryad at https://doi.org/10.5061/dryad.pzgmsbd0t.
