# Supplementary material for: Interactive Effects of Warming and Competition Do Not Limit the Adaptive Plastic Response to Drought in Populations of a Mediterranean Plant
Source: Glob Chang Biol. 2025 Jul 25;31(7):e70363. doi: 10.1111/gcb.70363 (PMC12291030; doi:10.1111/gcb.70363)
Supplement: Supplementary file 1 — Data S1. [file GCB-31-e70363-s001.pdf]

Supplementary figures and table

Figure S1. a) Location of sampled populations (green squares) and worldwide distribution of *Helianthemum squamatum* (yellow dots; data extracted from GBIF.org (23 June 2020); GBIF Occurrence Download https://doi.org/10.15468/dl.twuds9); b) General view of a population of *H. squamatum*, in the southwest of the Iberian Peninsula (SAX, Alicante); c) Adult and reproductive individual of *H. squamatum* in natural conditions in Belinchón population (BEL; Cuenca).

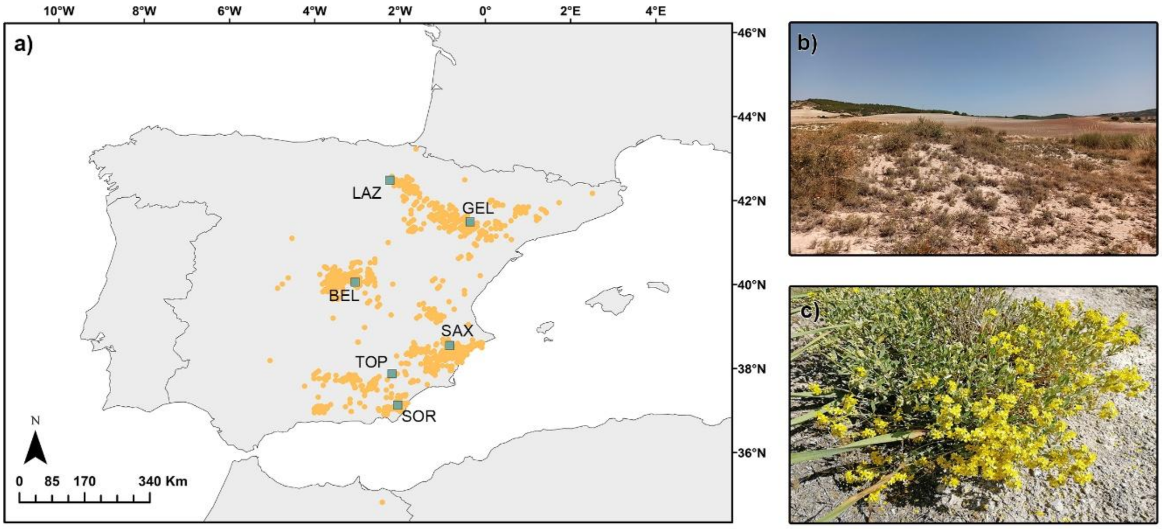

Figure S2. Experimental individuals growing below rain exclusion structures with and without Open Top Chambers (panels a) and b), respectively). Detailed pictures of experimental plants growing with and without an intraspecific competitor (panels c) and d), respectively).

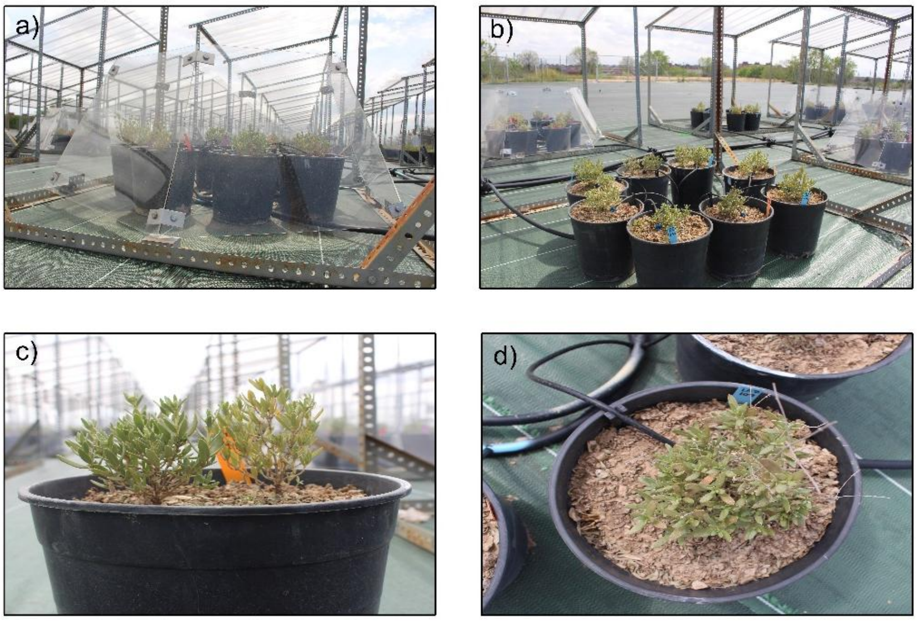

Figure S3. Soil water content (%) in pots of each of the eight experimental treatments. Mean values and standard errors in each treatment are shown throughout the experiment (from early March to early July 2022). Soil water content was monitored three days per week in 10-12 pots per treatment, using an HH2 Moisture Meter with an ML3 Sensor (Delta-T Devices, Cambridge, UK). Plants in the Well-watered level were kept at ~100% of field capacity for our gypsum soil (~24-26% of soil water content), and plants in the Drought level were maintained at ~50% of field capacity (~12-14% of soil water content). DR: Drought; WW: Well-watered; W: Warming, NW: No warming; NC: No competitor; C: Competitor.

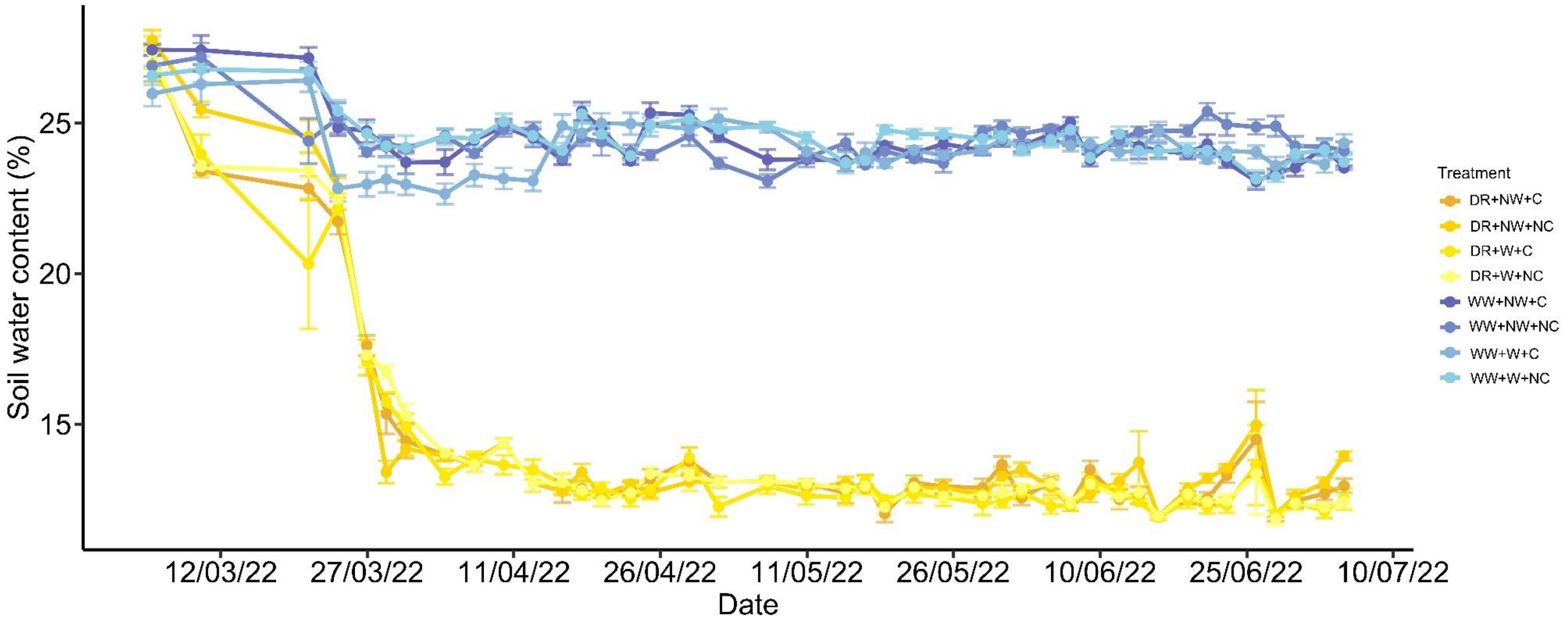

Figure S4. a) Air temperature; and b) relative humidity c) vapour pressure deficit (VPD) recorded throughout the experiment. Environmental conditions were monitored both within (in orange) and outside (in purple) OTCs by using 10 automated sensors that were placed randomly throughout the experimental set-up, in five pairs of two consecutive rain exclusion structures with and without OTC. Data were collected with HOBO U23 Pro v.2 Temp/RH (Onset Corporation, Bourne, MA, USA). To improve data visualization, the plot represents daily means instead of the 10-minute interval temperature and relative humidity data recorded. Different lines show the daily means of each of the ten sensors (five sensors within and five sensors outside OTCs). Mean temperature within and outside OTCs differed ~2.5 ºC, while relative humidity was slightly lower within OTCs (45.40 % ± 0.49) compared to outside (47.8 % ± 0.42), as well as VPD (2.58 ± 2.59 kPa under OTC conditions and 2.02 ± 1.91 kPa outside the OTCs).

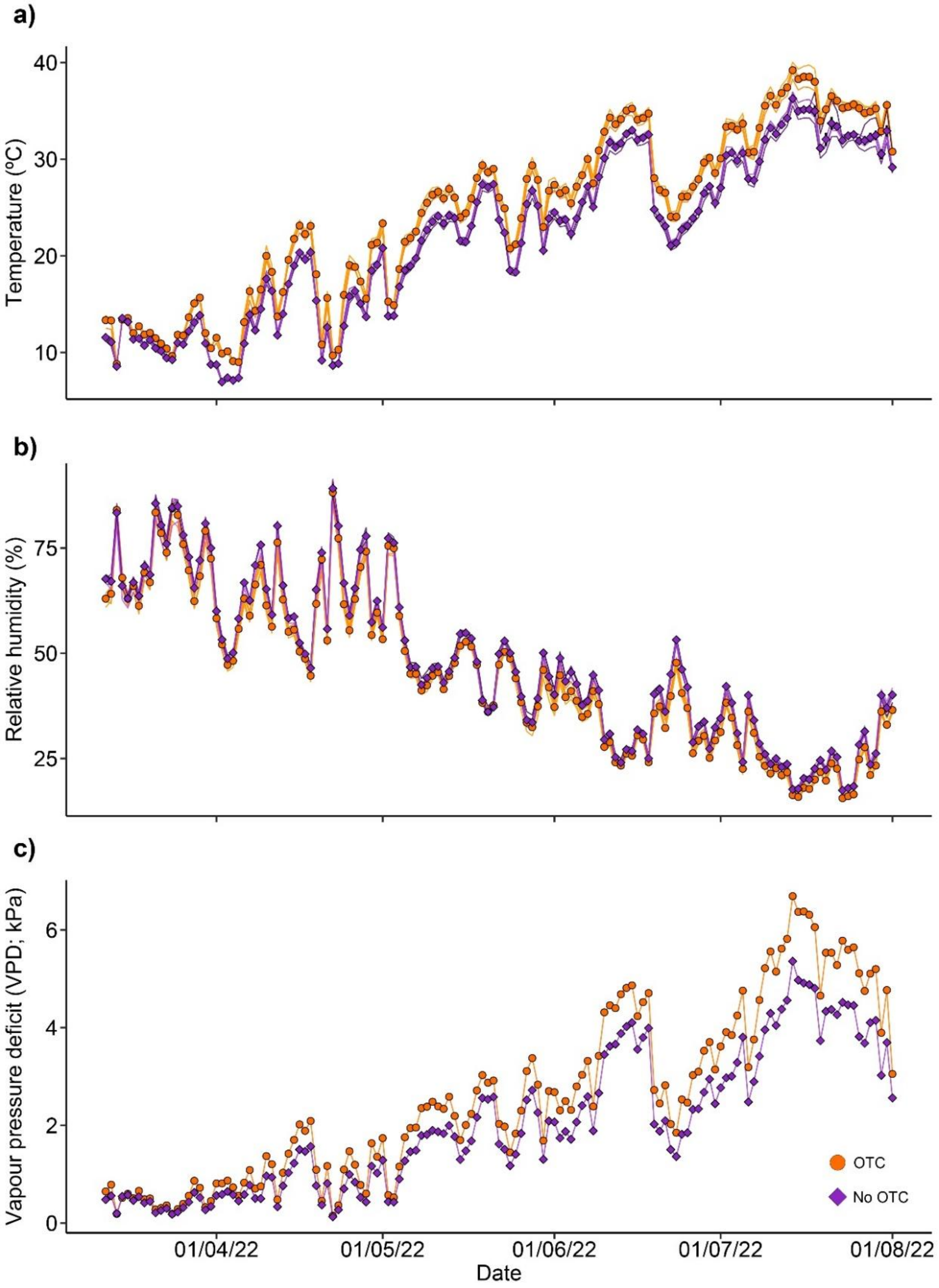

Table S1. Population location, code, coordinates and climatic conditions of the six populations of *Helianthemum squamatum* used in this study, the population where seeds from conspecific competitors were collected (Yebra, Guadalajara), and the outdoor CULTIVE facilities where the common garden experiment was performed. Climatic data of sampled populations was extracted from WorldClim bioclimatic layers using a 2km buffer around each population (Fick and Hijmans, 2017).

Population location	Pop. code	Long.	Lat.	Annual Precip. (mm)	Mean Temp. (ºC)	Max Temp. (ºC)	Min Temp. (ºC)
Topares (Almería)	TOP	-2.19	37.88	450.67	12.39	22.1	4.26
Belinchón (Cuenca)	BEL	-3.06	40.08	427.35	14.09	23.55	6.03
Lazagurría (Navarra)	LAZ	-2.23	42.48	461.75	13.23	20.93	6.35
Gelsa (Zaragoza)	GEL	-0.35	41.49	381.22	15.38	24.18	7.30
Sax (Alicante)	SAX	-0.85	38.55	353.89	15.08	23.53	7.65
Sorbas (Almería)	SOR	-2.12	37.1	291.0	16.45	24.46	9.52
Yebra (Guadalajara)	YEB	-2.94	40.35	432.0	13.8	22.95	6.02
CULTIVE (URJC)	CUL	-3.88	40.33	395.00	14.47	23.55	6.68

Table S2: Results of linear mixed models testing the effects of Population (P; df = 5), Water availability (W; df = 1), Temperature (T; df = 1), Intraspecific competition (C; df = 1) and their interactions (double, triple and quadruple interactions; df = 5 and 1 for interactions containing and not containing the term population, respectively) on all ecophysiological traits in *Helianthemum squamatum*. F-statistics are shown for each term. Significant terms are shown in bold (* P < 0.05; ** P < 0.01; *** P < 0.001) while † indicates a marginally significant effect (in italics; 0.05 < P < 0.1). R^2_m = marginal R^2; R^2_c = conditional R^2. See text for details on statistical analysis. SLA: Specific leaf area (cm^2/g); LDMC: Leaf dry matter content (mg/g); FO: Flowering onset (days); F_v/F_m: Midday photochemical efficiency; $\delta^{13}C$: Leaf Carbon isotope ratio (‰); $\delta^{15}N$: Leaf Nitrogen isotope ratio (‰); %N: Leaf nitrogen content; %C: Leaf carbon content; RGR: Relative growth rate (cm^3/days); ISM: Individual seed mass (mg); TSM: Total seed mass (mg); RB: Reproductive biomass (g).

	SLA	LDMC	FO	F_v/F_m	δ13C	δ15N	%N	%C	RGR	ISM	TSM	RB
	F	F	F	F	F	F	F	F	F	F	F	F
**Population (P)**	**11.45** ***	**30.488** ***	**19.688** ***	**2.719** *	**19.417** ***	**8.297** ***	**16.962** ***	**29.491** ***	**15.933** ***	**62.969** ***	**10.75** ***	**48.461** ***
**Water availability (W)**	**197.419** ***	**306.846** ***	**8.232** **	1.596	**424.301** ***	**83.412** ***	**131.428** ***	**46.741** ***	**24.572** ***	**48.462** ***	**206.98** ***	**520.919** ***
**Temperature (T)**	**25.303** ***	**26.98** ***	**661.812** ***	**92.956** ***	**9.476** **	**11.187** ***	**5.079** *	**25.984** ***	**6.715** *	**157.869** ***	**33.50** ***	**36.744** ***
**Intraspecific competition (C)**	1.413	**34.601** ***	**54.22** ***	0.024	**16.931** ***	2.674	**25.115** ***	**17.511** ***	**5.815** *	**9.005** **	**136.38** ***	**226.717** ***
**P×W**	1.328	**7.283** ***	2.549	0.462	1.423	2.288	2.36	**4.829** **	0.367	1.646	0.79	1.545
**P×T**	1.882	1.688	3.547	1.577	1.095	0.595	0.77	0.067	2.166	1.847	1.05	**3.285** *
**P×C**	1.181	0.809	**3.906** *	1.128	0.586	0.188	0.549	1.177	1.282	0.916	0.49	1.239
**W×T**	0.304	*5.087* †	1.701	0.257	0.043	2.893	**31.777** ***	*5.007* †	0.325	**6.949** *	0.79	**18.016** ***
**W×C**	0.473	0.212	0.909	2.99	**8.296** *	0.528	0.028	0.026	1.393	0.24	**7.31** **	**13.001** **
**C×T**	0.435	0.06	1.503	0.446	2.071	0.711	0.411	0.094	**8.154** *	0.089	**7.07** *	**7.612** **
**P×W×T**	1.049	0.963	0.787	0.636	1.005	0.37	0.574	1.828	2.327	1.964	2.37	**3.208** *
**P×W×C**	1.621	1.686	0.444	2.108	2.671	1.435	1.177	1.976	1.358	2.159	1.85	1.777
**P×C×T**	0.913	1.689	0.859	0.537	1.119	0.812	1.369	0.6	0.383	0.961	0.81	0.59
**W×C×T**	2.024	0.643	0.12	3.064	1.268	0.852	0.019	0.2	0.029	2.258	0.39	1.142
**P×W×C×T**	2.003	2.516	0.695	2.401	1.756	1.558	0.93	1.764	0.307	2.697	2.55	2.195
**R^2m/ R^2c**	0.332 0.349	0.466 0.474	0.57 0.58	0.184 0.224	0.516 0.531	0.242 0.264	0.359 0.37	0.35 0.395	0.275 0.331	0.502 0.505	0.402 0.551	0.651 0.666
